# New Unnatural Gallotannins: A Way toward Green Antioxidants, Antimicrobials and Antibiofilm Agents

**DOI:** 10.3390/antiox10081288

**Published:** 2021-08-13

**Authors:** Zuzana Hricovíniová, Šárka Mascaretti, Jana Hricovíniová, Alois Čížek, Josef Jampílek

**Affiliations:** 1 Institute of Chemistry, Slovak Academy of Sciences, Dúbravská Cesta 9, 845 38 Bratislava, Slovakia; 2 Regional Centre of Advanced Technologies and Materials, Czech Advanced Technology and Research Institute, Palacky University, Slechtitelu 27, 783 71 Olomouc, Czech Republic; sarka.mascaretti@gmail.com (Š.M.); josef.jampilek@gmail.com (J.J.); 3 Cancer Research Institute, Biomedical Research Center, Slovak Academy of Sciences, Dúbravská Cesta 9, 845 05 Bratislava, Slovakia; jana.hricoviniova@savba.sk; 4 Department of Infectious Diseases and Microbiology, Faculty of Veterinary Medicine, University of Veterinary Sciences Brno, Palackého 1946/1, 612 42 Brno, Czech Republic; cizeka@vfu.cz; 5 Department of Analytical Chemistry, Faculty of Natural Sciences, Comenius University, Ilkovičova 6, 842 15 Bratislava, Slovakia

**Keywords:** unnatural gallotannins, antioxidant potential, *S. aureus*, antibiofilm activity, antimicrobial effect

## Abstract

Nature has been a source of inspiration for the development of new pharmaceutically active agents. A series of new unnatural gallotannins (GTs), derived from d-lyxose, d-ribose, l-rhamnose, d-mannose, and d-fructose have been designed and synthesized in order to study the protective and antimicrobial effects of synthetic polyphenols that are structurally related to plant-derived products. The structures of the new compounds were confirmed by various spectroscopic methods. Apart from spectral analysis, the antioxidant activity was evaluated by 1,1-diphenyl-2-picrylhydrazyl (DPPH) radical-scavenging and iron reducing power (FRAP) assays. Antibacterial activity of compounds was tested in vitro against *Staphylococcus aureus* ATCC 29213, *Enterococcus faecalis* ATCC 29212 (reference and control strains), three methicillin-resistant isolates of *S. aureus*, and three isolates of vancomycin-resistant *E. faecalis*. For screening of antimycobacterial effect, a virulent isolate of *Mycobacterium tuberculosis* and two non-tuberculous mycobacteria were used. Furthermore, antibiofilm activity of structurally different GTs against *S. aureus*, and their ability to inhibit sortase A, were inspected. Experimental data revealed that the studied GTs are excellent antioxidants and radical-scavenging agents. The compounds exhibited only a moderate antibacterial effect against Gram-positive pathogens *S. aureus* and *E. faecalis* and were practically inactive against mycobacteria. However, they were efficient inhibitors and disruptors of *S. aureus* biofilms in sub-MIC concentrations, and interacted with the quorum-sensing system in *Chromobacterium* *violaceum*. Overall, these findings suggest that synthetic GTs could be considered as promising candidates for pharmacological, biomedical, consumer products, and for food industry applications.

## 1. Introduction

Tannins are a large sub-class of polyphenolic compounds ubiquitously present in plants. They are found in a variety of species, playing roles in the plant’s natural defence system against environmental stressors and microbial infections [1,2,3]. Natural tannins are widely studied for their prophylactic and therapeutic potential [4,5]. Gallotannins (GTs) from various species have been extensively studied as they exhibit multiple biological activities ranging from antioxidant, radical scavenging, antimicrobial, anti-inflammatory, and immune-modulatory to anticancer effects [2,6,7,8,9]. Moreover, numerous plant polyphenols have exhibited strong antibacterial and antibiofilm activity against staphylococci [10,11]. Among the staphylococci, *Staphylococcus aureus* is of most clinical concern. Undesirable bacterial *S. aureus* biofilm layers are formed on indwelling medical devices or food processing contact-surfaces, resulting in microbial communities more resistant to the traditional disinfectants [12,13,14].

The molecular structure of GTs is generally composed of a central carbohydrate core esterified with gallic acid (GA). Structurally related polyphenolic compounds, e.g., 1,2,3,6-tetra-*O*-galloyl-d-galactose [15], 1-*O*-galloyl-l-rhamnose [16], 7-*O*-galloyl- d-sedoheptulose [17], 1,2,3,4,6-penta-*O*-galloyl-d-glucose [7,18,19], 2,3-di-*O*-galloyl- d-glucitol, or 2,3,6-tri-*O*-galloyl-d-glucitol [20], exert interesting biological activities. Among the vast number of bioactive polyphenols, 1,2,3,4,6-penta-*O*-galloyl-d-glucose (PGG), has been the most widely studied. A number of in vitro and in vivo studies have shown that PGG exhibits diverse pharmacological effects [7,19,21]. Interesting anti-staphylococcal activity was observed for PGG isolated from the Thai mango (*Mangifera indica* L.). The antibacterial investigation of a crude GT extract on *S. aureus* revealed that PGG was the most effective component [22]. The effect of the extract was also synergistic with penicillin G. Damaging effects on the cell membrane, leading to an alteration in cell morphology and interference with bacterial division, were suggested as a possible inhibitory mechanism. Moreover, the PGG exhibited a remarkable anti-biofilm activity. It was observed that PGG noticeably inhibited the initial phase of biofilm formation of *S. aureus* [23]. Natural PGG, isolated from *Paeonia suffruticosa*, was evaluated for its antifungal activity in vitro, against *Candida glabrata*. According to the MIC values, PGG was 10-fold more effective than the standard antifungal drug fluconazole. It was demonstrated that the antifungal activity of PGG is mediated by local ruptures in the cell wall, but that these did not affect plasmatic membrane, nucleus or mitochondria [24].

Galloylated branched-chain sugars, are a rare class of naturally occurring GTs. A typical example is 2′,5-di-*O*-galloyl-2-*C*-(hydroxymethyl)-d-ribose (hamamelitannin) which is an active component of various plant extracts [25,26,27]. The antioxidant and radical scavenging effect of hamamelitannin have been studied, and the respective molecular mechanisms were described in detail [28,29,30]. Natural hamamelitannin has also been reported as efficient antiviral, antibacterial, antibiofilm, anti-inflammatory, and anticancer agent [31,32,33,34].

The biological effects of galloylated branched-chain sugars have not been investigated in detail due to the difficulties with isolation of the pure compounds from plants. Solution toward this end is the synthesis of naturally identical compounds or their analogues. As a part of our ongoing studies on biologically important sugars as potential drug candidates, we have designed and synthesized new galloyl-derivatives of 2-*C*-hydroxymethyl-branched sugars derived from d-lyxose, d-ribose, d-mannose, l-rhamnose, d-fructose and compared their biological activities with the unnatural GTs (methyl tetra-*O*-galloyl-α-d-glucoside, methyl tetra-*O*-galloyl-α-d-mannoside, methyl tri-*O*-galloyl-α-l-rhamnoside), penta-*O*-galloyl-d-glucose, and gallic acid. The effects of GTs (with a degree of galloylation ranging from 1 to 5) on environmental and human pathogens were examined in various experimental systems. Structurally different GTs were screened in vitro for their antimicrobial properties against a spectrum of staphylococci, enterococci, and mycobacteria. Their ability to eradicate pre-formed bacterial biofilms of *S.* *aureus*, and quorum sensing (QS) inhibition in *Chromobacterium violaceum* was also examined.

## 2. Materials and Methods

### 2.1. Physico-Chemical Methods

High-resolution NMR spectra were recorded in a 5 mm cryoprobe on a Bruker Avance III HD spectrometer (Bruker, Karlsruhe, Germany) equipped with a CryoProbe (Bruker) at 25 °C in acetone-*d*_6_, methanol-*d*_4_ or chloroform-*d* (Sigma-Aldrich, St. Louis, MO, USA). The proton and carbon chemical shifts were referenced to tetramethylsilane (TMS). Optical rotations were determined at 20 °C with an automatic polarimeter PerkinElmer Model 141 (Waltham, MA, USA) using a 10 cm, 1 mL cell. High-resolution mass determination (HRMS) was performed by electrospray ionization mass spectrometry (ESI-MS) on an Orbitrap instrument (Thermo Scientific, West Palm Beach, FL, USA). The progress of the reactions was monitored by thin layer chromatography (TLC) on Merck silica gel 60 or silica gel 60 F254 glass plates (Merck, Darmstadt, Germany). Flash column chromatography was performed with Silica gel (40–100 μm) (Merck Milipore, Burlington, MA, USA). Solvent A (ethyl acetate/hexane/methanol, 6:1:1, *v*/*v*/*v*); solvent B (chloroform/methanol 8:2 *v/v*); solvent C (ethyl acetate/cyclohexane/hexane, 4:3:1, *v*/*v*/*v*); solvent D (ethyl acetate/hexane, 6:1, *v*/*v*); and solvent E (ethyl acetate/hexane, 3:1, *v*/*v*). Gallic acid (GA), methyl gallate, *N*,*N*-dicyclohexylcarbodiimide (DCC), 4-dimethylaminopyridine (DMAP), 1,2:4,5-di-*O*-isopropylidene-d-fructopyranose, 1,1-diphenyl-2-picrylhydrazylradical (DPPH), potassium ferricyanide, and 3-(4,5-dimethylthiazol-2-yl)-2,5-diphenyltetrazolium bromide (MTT) (Sigma-Aldrich) were commercial products. All other chemicals and solvents were of analytical grade and were used without further purification.

### 2.2. Studied Compounds

Methyl 2,3,4,6-tetra-*O*-galloyl-α-d-glucoside (G_4_Glc), methyl 2,3,4,6-tetra-*O*-galloyl- α-d-mannoside (G_4_Man), methyl 2,3,4-tri-*O*-galloyl-α-l-rhamnoside (G_3_Rham) were synthesized by Hricovíniová et al., and all spectral data for these GTs were reported in our previous work [35]. 1,2,3,4,6-penta-*O*-galloyl-αβ-d-glucose (PGG) was synthesized according to Ren et al. [18] and its identity and purity was confirmed by NMR spectroscopy. ^1^H NMR (600.13 MHz, methanol-*d*_4_) *δ*: 7.21, 7.11, 7.11, 7.05, 6.99, 6.98, 6.95,6.93, 6.92, 6.90 (10 × s, 20 H, galloyl), 6.70 (d, 1H, *J*_1,2_ = 3.5 Hz, H-1α), 6.24 (d, 1H, *J*_1,2_ = 8.3 Hz, H-1β), 6.13 (dd, 1H, *J*_3,4_ = 9.9 Hz, H-3α), 5.90 (dd, 1H, *J*_3,4_ = 9.9 Hz, H-3β), 5.71 (dd, 1H, *J*_4,5_ = 10.2 Hz, H-4α), 5.62 (dd, 1H, *J*_4,5_ = 10.1 Hz, H-4β), 5.59 (dd, 1H, *J*_2,3_ = 9.7 Hz, H-2β), 5.50 (dd, 1H, *J*_2,3_ = 10.1 Hz, H-2α), 4.57 (m, 1H, H-5α), 4.52 (m, 2H, H-6β), 4.47 (m, 2H, H-6α), 4.41 (m, 1H, H-5β); ^13^C NMR (150.91 MHz, methanol-*d*_4_) *δ*: 168.08–166.11 (C=O), 146.93–146.43 (CH-Ar), 93.97 (C-1β), 91.13 (C-1α), 74.58 (C-5β), 74.26 (C-3β), 72.35 (C-2β), 72.19 (C-5α), 71.77 (C-2α), 71.74 (C-3α), 69.96 (C-4β), 69.78 (C-4α), 63.27 (C-6β), 63.18 (C-6α).

### 2.3. Synthesis of Galloyl Derivatives of 2-C-(Hydroxymethyl)-branched Saccharides

#### 2.3.1. Preparation of 2-*C*-(Hydroxymethyl)-2,3-*O*-isopropylidene-α-d-lyxofuranose (**1**)

A reaction mixture of 2,3-*O*-isopropylidene-d-lyxose [36], (1 g; 5.3 mmol), K_2_CO_3_ (0.8 g) methanol (15 mL), and 37% aqueous solution of formaldehyde (15 mL; 147 mmol) was refluxed in argon atmosphere at 85 °C for 40 h. The reaction mixture was neutralized with 10% aq sulphuric acid and evaporated. Extraction with chloroform (4 × 30 mL) gave a combined fraction that was dried over anhydrous MgSO_4_ and concentrated. Crude reaction mixture was purified by column chromatography on silica gel (solvent A). Yield 82%; R_f_ = 0.58 (solvent A); ${\left[\alpha \right]}_{d}^{20}$ = +3.33→4.6° (c 1, acetone); HRMS: calcd for C_9_H_16_O_6_Na [M+Na]^+^ 243.0845; found 243.0914 [M+Na]^+^; ^1^H NMR (600.13 MHz, acetone-*d*_6_) *δ*: 5.24 (s, 1H, H-1), 4.61(d, 1H, H-3), 4.19 (d, 1H, H-4), 3.83 (m, 2H, C*H*-2a, C*H*-5a), 3.73 (2H, C*H*-2b, C*H*-5b),1.46, 1.40 (2 × s, 6H, CH_3_(Ip)); ^13^C NMR (150.91 MHz, acetone-*d*_6_) *δ*: 104.73 (C-1), 96.14 (C-2), 84.47 (C-4), 82.50 (C-3), 63.52 (*C*H_2_(C-2)), 61.45 (*C*H_2_(C-5), 29.42, 29.33 (2 × s *C*H_3_(Ip).

#### 2.3.2. Preparation of 2-*C*-(Hydroxymethyl)-2,3-*O*-isopropylidene-α-d-lyxofuranose (**2**)

The 3,4,5-tri-*O*-benzyl protected gallic acid was synthesized from methyl gallate by the method reported previously [35]. Recrystallization from chloroform afforded white cotton-like needles, m.p. 198–199 °C [37]. ^1^H NMR (600.13 MHz, acetone-*d*_6_) *δ*: 7.27–7.55 (m, 17H, Ar-H), 5.23 (s, 4H, 2 PhC*H*_2_), 5.14 (s, 2H, PhC*H*_2_).

#### 2.3.3. Preparation of Di-*O*-(3,4,5-tri-*O*-benzylgalloyl)-2-*C*-(hydroxymethyl)-2,3-*O*-isopro- pylidene Aldoses

The reaction mixture of 2,3-*O*-isopropylidene derivative of 2-*C*-(hydroxymethyl)-d-lyxose (**1**), 2,3-*O*-isopropylidene-2-*C*-(hydroxymethyl)-d-ribose [38], or 2,3-*O*-isopropylidene 2-*C*-(hydroxymethyl)-l-rhamnose [39] (1 equiv), DMAP (2 equiv), and **2** (2 equiv) in dry dichloromethane (25 mL) was refluxed for 1 h under argon atmosphere. After that DCC (2 equiv) was added and stirring continued at r.t. for 18 h. Then, the reaction mixture was placed into the ice-bath for 1 h and the precipitate was filtered. The filtrate was washed with 3% aq HCl (1 × 30 mL), saturated NaHCO_3_ (1 × 30 mL), and brine (1 × 30 mL). The combined organic layer was dried over anhydrous MgSO_4_, evaporated, and flash column chromatography on silica gel afforded the desired products (**3**–**5**).

*2′,5-Di-O-(3,4,5-tri-O-benzylgalloyl)-2-C-(hydroxymethyl)-2,3-O-isopropylidene-d-lyxofuranose* (**3**). Yield 73%; ${\left[\alpha \right]}_{d}^{20}$ = +21.0 (c 1, CHCl_3_); R_f_ = 0.95 (solvent B); HRMS: calcd for C_65_H_60_O_14_Na [M+Na]^+^ 1087.1560; found 1087.1584 [M+Na]^+^; ^1^H NMR (600.13 MHz, chloroform-*d*) *δ*: 7.48–7.14 (m, H-Ar), 6.58 (s, 1H, H-1), 4.79 (m, 1H, H-5a), 4.75 (d, 1H, H-2′a), 4.71 (m, 2H, H-3), 4.48 (m, 1H, H-5b), 4.46 (d, 1H, H-2′b), 4.42 (m, 1H, H-4), 1.59, 1.43 (2 × s, 6H, CH_3_(Ip)); ^13^C NMR (150.91 MHz, chloroform-*d*) *δ*: 128.52–127.25 (CH Ar), 101.25 (C-1), 93.51 (C-2), 82.80 (C-3), 80.35 (C-4), 64.34 (*C*H_2_(C-2)), 62.51 (*C*H_2_(C-5)).

*2′,5-Di-O-(3,4,5-tri-O-benzylgalloyl)-2-C-(hydroxymethyl)-2,3-O-isopropylidene-d-ribofuranose* (**4**). Yield 69%; ${\left[\alpha \right]}_{d}^{20}$ = −13.5 (c 1, Ac); R_f_ = 0.95 (solvent B);HRMS: calcd for C_65_H_60_O_14_Na [M+Na]^+^ 1087.1560; found 1087.1577 [M+Na]^+^; ^1^H NMR (600.13 MHz, chloroform-*d*) *δ*: 7.38–7.04 (m, H-Ar), 6.72 (s, 1H, H-1), 4.72 (d, 1H, H-2′a), 4.67 (m, 2H, H-3, H-4), 4.52 (m, 1H, H-5a), 4.47 (d, 1H, H-2′b), 4.24 (m, 1H, H-5b), 1.65, 1.51 (2 × s, 6H, CH_3_(Ip)); ^13^C NMR (150.91 MHz, chloroform-*d*) *δ*: 127.48–126.31 (CH Ar), 101.24 (C-1), 92.29 (C-2), 84.16 (C-4), 83.57 (C-3), 63.89 (*C*H_2_(C-2)), 63.13 (*C*H_2_(C-5)).

*2′,5-Di-O-(3,4,5-tri-O-benzylgalloyl)-2-C-(hydroxymethyl)-2,3-O-isopropylidene-β-l-rhamnopyranose* (**5**). Yield 78%; ${\left[\alpha \right]}_{d}^{20}$ = −36.6 (c 1, Ac); R_f_ = 0.83 (solvent C); HRMS: calcd for C_66_H_62_O_14_Na [M+Na]^+^ 1101.4040; found 1101.4062 [M+Na]^+^; ^1^H NMR (600.13 MHz, acetone-*d*_6_) *δ*: 7.53–7.22 (m, H-Ar), 6.51 (s, 1H, H-1), 5.29 (m, 1H, H-5), 4.86 (m, 1H, H-3), 4.81 (d, 2H, H-2′a), 4.64 (d,1H, H-2′b), 4.39 (dd, 1H, H-4), 1.48, 1.32 (2 × s, 6H, CH_3_(Ip)), 1.38 (s, 3H, CH_3_(Ip)); ^13^C NMR (150.91 MHz, acetone-*d*_6_) *δ*: 128.75–127.42 (*C*H Ar), 101.14 (C-1), 93.60 (C-2), 83.98 (C-4), 82.55 (C-3), 68.42 (C-5), 65.29 (*C*H_2_(C-2)), 17.02 (CH_3_).

*2′-O-(3,4,5-tri-O-Benzylgalloyl)-2-C-(hydroxymethyl)-2,3:5,6-di-O-isopropylidene-d-mannofuranose* (**6**). The mixture of 2-*C*-(hydroxymethyl)-2,3:5,6-di-*O*-isopropylidene- d-mannofuranose [40] (1 equiv), DMAP (1 equiv), and **2** (1 equiv) in dry dichloromethane (25 mL) was refluxed for 1 h under argon atmosphere. After that, DCC (1 equiv) was added and stirring continued at r.t. overnight. The reaction mixture was worked up in exactly the same way as mentioned in previous experiments. Derivative **6** was separated by flash column chromatography on silica gel. Yield 75%; ${\left[\alpha \right]}_{d}^{20}$ = +53.0 (c 1, Ac); R_f_ = 0.85 (solvent C); HRMS: calcd for C_41_H_44_O_11_Na [M+Na]^+^ 735.2781; found 735.2815 [M+Na]^+^; ^1^H NMR (600.13 MHz, chloroform-*d*) *δ*: 7.58–7.47 (m, H-Ar), 6.50 (s, 1H, H-1), 4,73 (d, 1H, H-2′a), 4.71 (d, 1H, H-3), 4.48 (m, 1H, H-2′b), 4.47 (m, 1H, H-5), 4.13 (m, 1H, H-5′a), 4.03 (m, 1H, H-5′b), 4.05 (m, 1H, H-4),1.57, 1.44, 1.41, 1.38 (4 × s, 12H, CH_3_(Ip)); ^13^C NMR (150.91 MHz, chloroform-*d*) *δ*: 128.53–127.45 (*C*H Ar), 101.55 (C-1), 93.47 (C-2), 82.72 (C-3, C-4), 72.61 (C-5), 67.05 (*C*H_2_(C-5)), 64.49 (*C*H_2_(C-2)).

*3-O-(3,4,5-tri-O-Benzylgalloyl)-1,2:4,5-di-O-isopropylidene-d-fructopyranose* (**7**). The mixture of 1,2:4,5-di-*O*-isopropylidene-d-fructopyranose (1 equiv), DMAP (1 equiv), and **2** (1 equiv) in dry dichloromethane (25 mL) was refluxed for 1 h under argon atmosphere. After that, DCC (1 equiv) was added and stirring continued at r.t. overnight. The reaction mixture was worked up as mentioned in previous experiment. Yield 84%; ${\left[\alpha \right]}_{d}^{20}$ = −85.3 (c 1, CHCl_3_); R_f_ = 0.91 (solvent D); HRMS: calcd for C_40_H_42_O_10_Na [M+Na]^+^ 705.2676; found 705.2825 [M+Na]^+^; ^1^H NMR (600.13 MHz, chloroform-*d*) *δ*: 7.46–7.32 (m, H-Ar), 5.35 (d, 1H, H-3), 4.44 (d, 1H, H-4), 4.28 (d, 1H, H-5), 4.19 (dd, 1H, H-6a),4.14 (dd, 1H, H-6b),4.00 (dd, 1H, H-1a), 3.88 (dd, 1H, H-1b), 1.62 (s, 3H, CH_3_(Ip)), 1.51 (s, 3H, CH_3_(Ip)), 1.39 (s, 3H, CH_3_(Ip)), 1.37 (s, 3H, CH_3_(Ip)); ^13^C NMR (150.91 MHz, chloroform-*d*) *δ*: 128.56–127.52 (*C*H Ar), 112.11 (1,2*C*Me_2_), 109.77 (4,5*C*Me_2_), 75.10 (C-4), 73.82 (C-5), 71.72 (C-1), 70.80 (C-3), 60.49 (C-6), 27.87, 26.47, 26.40, 26.29 (4 × CH_3_(Ip)).

#### 2.3.4. Debenzylation Procedure

A solution of *O*-benzylgalloyl-derivative **3**–**7** (100 mg) in methanol (6 mL) was stirred for 16 h in the presence of 10% Pd/C (25 mg) in hydrogen atmosphere. The reaction mixture was filtered, washed with methanol (1 × 10 mL) and concentrated in vacuo to give respective per-*O*-galloyl-derivatives **8**–**12** as amorphous solids.

*2′,5-Di-O-galloyl-2-C-(hydroxymethyl)-2,3-O-isopropylidene-d-lyxofuranose* (**8**, G_2_Lyx). Yield 90%; ${\left[\alpha \right]}_{d}^{20}$ = +29.0 (c 1, CHCl_3_); R_f_ = 0.73 (solvent A); HRMS: calcd for C_23_H_24_O_14_Na [M+Na]^+^ 547.1064; found 547.1085 [M+Na]^+^; ^1^H NMR (600.13 MHz, methanol-*d*_4_) *δ*: 7.03, 7.01 (2 × s, 4H, galloyl), 6.72 (s, 1H, H-1), 4.94 (d, 1H, H-3), 4.71, 4.66 (m, 2H, H-2′a, H-2′b), 4.60 (m, 1H, H-5′a), 4.58 (m, 1H, H-4), 4.44 (m, 1H, H-5′b), 1.57, 1.42 (2 × s, 6H, CH_3_(Ip)); ^13^C NMR (150.91 MHz, methanol-*d*_4_) *δ*: 111.52, 111.43 (2 × CH, galloyl), 103.65 (C-1), 96.39 (C-2), 85.44 (C-3), 82.80 (C-4), 65.61 (*C*H_2_(C-2)), 64.68 (*C*H_2_(C-5)).

*2′,5-Di-O-galloyl-2-C-(hydroxymethyl)-2,3-O-isopropylidene-d-ribofuranose* (**9**, G_2_Rib). Yield 93%; ${\left[\alpha \right]}_{d}^{20}$ = −49.6 (c 1, Ac); R_f_ = 0.54 (solvent D); HRMS: calcd for C_23_H_24_O_14_Na [M+Na]^+^ 547.1064; found 547.1079 [M+Na]^+^; ^1^H NMR (600.13 MHz, methanol-*d*_4_) *δ*: 7.04, 7.03 (2 × s, 4H, galloyl), 6.50 (s, 1H, H-1), 4.91 (d, 1H, H-3), 4.68 (m, 2H, H-2′a, H-2′b), 4.56 (m, 1H, H-4), 4.44 (m, 1H, H-5′a), 4.32 (m, 1H, H-5′b), 1.54, 1.44 (2 × s, 6H, CH_3_(Ip)); ^13^C NMR (150.91 MHz, methanol-*d*_4_) *δ*: 108.87 (2 × CH, galloyl), 101.90 (C-1), 93.28 (C-2), 85.40 (C-4), 84.65 (C-3), 63.87 (*C*H_2_(C-5)), 63.65 (*C*H_2_(C-2)).

*2′,4-Di-O-galloyl-2-C-(hydroxymethyl)-2,3-O-isopropylidene-β-l-rhamnopyranose* (**10**, G_2_Rham). Yield 95%; ${\left[\alpha \right]}_{d}^{20}$ = −60.5 (c 1, MeOH); R_f_ = 0.84 (solvent A); HRMS: calcd for C_24_H_26_O_14_Na [M+Na]^+^ 561.1220; found 561.1196 [M+Na]^+^; ^1^H NMR (600.13 MHz, methanol-*d*_4_) *δ*: 7.07, 7.04 (2 × s, 4H, galloyl), 6.33 (s, 1H, H-1), 5.23 (m, 1H, H-5), 4.91 (d, 1H, H-3), 4.60 (m, 2H, H-2′a, H-2′b), 4.36 (dd, 1H, H-4), 1.56, 1.38 (2 × s, 6H, CH_3_(Ip)), 1.40 (s, 3H, CH_3_(Ip)); ^13^C NMR (150.91 MHz, methanol-*d*_4_) *δ*: 109.27, 109.07 (2 × CH, galloyl), 100.52 (C-1), 93.65 (C-2), 83.77 (C-4), 82.43 (C-3), 67.52 (C-5), 63.27 (*C*H_2_(C-2)), 17.06 (CH_3_).

*2′-O-Galloyl-2-C-(hydroxymethyl)-2,3:5,6-di-O-isopropylidene-d-mannofuranose* (**11**, GMan). Yield 89%; ${\left[\alpha \right]}_{d}^{20}$ = +16.3 (c 1, MeOH); R_f_ = 0.52 (solvent B); HRMS: calcd for C_20_H_26_O_11_Na [M+Na]^+^ 465.1373; found 465.1396 [M+Na]^+^; ^1^H NMR (600.13 MHz, acetone-*d*_6_) *δ*: 7.16 (s, 2H, galloyl), 4.70 (d, 1H, H-3), 4.65 (s, 1H, H-1), 4.55 (d, 1H, H-2′a), 4.37 (m, 1H, H-5), 4.33 (d, 1H, H-2′b), 4.06 (m, 1H, H-5′a), 4.01 (m, 1H, H-5′b), 3.94 (m, 1H, H-4), 1.47, 1.37, 1.35, 1.29 (4 × s, 12H, CH_3_(Ip)); ^13^C NMR (150.91 MHz, acetone-*d*_6_) *δ*: 109.93 (*C*H, galloyl), 105.50 (C-1), 90.67 (C-2), 82.21 (C-3), 78.90 (C-4), 74.35 (C-5), 67.28 (*C*H_2_(C-5)), 64.66 (*C*H_2_(*C*-2)).

*3-O-Galloyl-1,2:4,5-di-O-isopropylidene-d-fructopyranose* (**12**, GFru). Yield 93%; ${\left[\alpha \right]}_{d}^{20}$ = −102.9 (c 1, Ac); R_f_ = 0.75 (solvent E); HRMS: calcd for C_19_H_24_O_10_Na [M+Na]^+^ 435.1267; found 435.1304 [M+Na]^+^; ^1^H NMR (600.13 MHz, acetone-*d*_6_) *δ*: 7.19 (s, 2H, galloyl), 5.37 (d, 1H, H-3), 4.06 (dd, 1H, H-4), 4.01 (dd, 1H, H-6a), 3.99 (m, 1H, H-5),3.86 (dd, 2H, H-1a, H-1b), 3.76 (dd, 1H, H-6b),1.43, 1.37, 1.34, 1.29 (4 × s, 12H, CH_3_(Ip)); ^13^C NMR (150.91 MHz, acetone-*d*_6_) *δ*:110.33 (*C*H, galloyl), 72.60 (C-1), 70.92 (C-3), 70.59(C-5), 70.15 (C-4), 65.46 (C-6), 26.97, 26.84, 26.56, 26.24 (4 × CH_3_(Ip)).

### 2.4. Determination of Antioxidant Activity 

#### 2.4.1. 1,1-Diphenyl-2-picrylhydrazyl Radical Scavenging Activity (DPPH Assay)

The free radical-scavenging capacity was evaluated by DPPH assay [41] with some modifications [35]. The synthesized per-*O*-galloyl-derivatives of different concentrations (0.1–1 mM) were dissolved in methanol. The reaction mixture consisting of the tested sample (50 µL) and DPPH solution (950 µL) was added to 96-well plate incubated (30 min) and absorbance was measured at 517 nm using an xMark^TM^ Microplate Spectrophotometer (Bio-Rad Laboratories Inc., Hercules, CA, USA). GA and DPPH solution were used as a positive and negative control. The reaction was conducted at r.t. and experiments were performed in triplicate. The radical-scavenging activity of tested compounds was calculated using the equation:

DPPH radical scavenging activity (%) = 100 × (*A*_0_ − *A*_S_)/*A*_0_

where *A*_0_ is the absorbance of the negative control, and *A*_S_ is the absorbance of the tested compound.

#### 2.4.2. Iron(III)-Reducing Power Assay (FRAP Assay)

The reducing activity of compounds was evaluated by colorimetric FRAP assay [42] with some modifications described previously [35]. Methanolic solution (200 µL) of tested compounds (0.1–1 mM) were mixed with 0.2 M phosphate buffer (500 µL, pH 6.6) and 1% potassium ferricyanide [K_3_Fe(CN)_6_] (500 µL) and incubated at 50 °C for 20 min. The absorbance was recorded at 700 nm using an xMark^TM^ Microplate Spectrophotometer.

### 2.5. Biological Studies

#### 2.5.1. Test Microorganisms

Standard reference bacterial strains *Staphylococcus aureus* ATCC 29213, *Enterococcus faecalis* ATCC 2921, *Mycobacterium tuberculosis* H_37_Ra, and *Chromobacterium violaceum* ATCC 12472 were obtained from American Type Culture Collection (ATTC). Three vancomycin-resistant isolates of *E. faecalis* (VRE 342B, VRE 368, VRE 725B) were provided by Oravcová et al. [43]. Clinical isolates of methicillin-resistant *S. Aureus* (MRSA 63718, MRSA 630, MRSA3202) and non-tuberculoid mycobacteria (*Mycobacterium smegmatis* and *Mycobacterium kansasii*) were obtained from a collection of the Department of Infectious Diseases and Microbiology, University of Veterinary Sciences Brno (Czech Republic).

#### 2.5.2. Determination of Antimicrobial Activity 

Antimicrobial activity of compounds was tested using microtitration broth method according to Clinical and Laboratory Standards Institute [44] with some modifications described previously [45,46,47]. Compounds were dissolved in DMSO to get concentration 10 mg/mL and diluted in a microtitration plate in an appropriate medium, to reach the final concentration 256–2 µg/mL. Subsequently, a microtitration plate was inoculated with tested microorganisms. The minimum inhibitory concentration (MIC) was evaluated visually as the minimal concentration of tested compound, which completely inhibited the bacterial growth. Each experiment was repeated at least three times.

#### 2.5.3. Biofilm Inhibition 

The ability of compounds to prevent biofilm growth was tested according to a previously described method [44,47]. Compounds were diluted in a 96-well microtitration plate in tryptic soya broth (TSB) containing 2% glucose to reach the final concentrations 256–2 µg/mL. The plates were inoculated with inoculum of *S. aureus* ATCC 29213 in logarithmic growth phase. The final concentration of bacterial cells in the wells was 1 × 10^5^ CFU/mL. The plates were incubated at 37 °C for 48 h, the contents of the wells were removed and washed with sterile phosphate buffered saline (PBS). After drying, 0.5% crystal violet was added to each well, and the plates were incubated at r.t. for 20 min. After that, the dye was removed and the plates were washed three times with sterile PBS. The coloured biofilm was detached from the surface using 33% solution of acetic acid (125 µL). The absorbance at 595 nm was measured (Tecan Infinite 200 PRO, Tecan, Grodig, Austria) and the percentage of inhibition was determined. As a blank, a non-inoculated plate treated in the same way was used [46,48]. The ability to inhibit biofilm formation was evaluated as a percentage inhibition of growth compared to the growth control according to the equation:

% of inhibition = 100 − (*OD*_595S_/*OD*_595C_) × 100

where *OD*_595S_ is the absorbance of the sample at 595 nm, and *OD*_595C_ is the absorbance of the growth control at 595 nm. The minimum biofilm inhibitory concentration was assessed as the lowest concentration of the tested compounds, which inhibited the growth of 80% bacteria compared to the growth control. The experiment was made in duplicate and was repeated at least three times.

#### 2.5.4. Biofilm Disruption

In order to test the ability of the studied GTs to eradicate a matured biofilm, a staphylococcus biofilm was prepared in the same way as for inhibitory activity testing. Biofilms were grown as described in previous experiment, but without the presence of the compounds. The matured biofilms were treated with compounds diluted in CaMH (100 µL) to concentration 256–2 µg/mL. A growth control containing 2.5% of DMSO in CaMH was included. The plates were incubated at 37 °C for 24 h. Bacterial viability was analysed by adding of MTT solution (0.5 mg/mL) in PBS (100 µL) to each well. The plates with MTT were incubated at 37 °C for 1–2 h in the dark, until the blue formazan crystals appeared. The solution in the wells was removed, and the plates were washed with PBS. The formazan crystals were dissolved by using 17% sodium dodecyl sulphate in 40% dimethylformamide [46,49]. The absorbance at 570 nm was measured and the percentage of eradication was determined according to the equation:

% of biofilm eradication = 100 − (*OD*_570S_/*OD*_570C_) × 100

where *OD*_570S_ is the absorbance of the sample at 570 nm, and *OD*_570C_ is the absorbance of the growth control at 570 nm. The minimum biofilm eradication concentration was the lowest concentration of the compound, which reduced the metabolic activity of the biofilm by 80% compared to the growth control. The experiment was made in duplicate and repeated at least three times.

#### 2.5.5. Interaction with Quorum Sensing of *C. violaceum*

*C. violaceum* was cultivated on Mueller-Hinton (MH) agar at r.t. for 48 h. Then, several colonies were suspended in Lysogeny broth medium (LB) and cultivated at r.t. overnight. This culture (50 µL) was mixed with dilute 0.1% MH agar (5 mL). The mixture was poured onto pre-prepared Petri dishes with MH agar. When the agar on the plates got solid, wells (diameter of 5 mm) were made sterile in the dish. The wells were at least 25 mm apart and from the edge. The test substance (20 µL) of the desired concentration, prepared from a stock solution of the substance in DMSO (10 mg/mL) diluted in PBS, was added into each well; DMSO blank served as the control. After that, the plates were incubated for 3 days at r.t. until there was a visual growth of *C. violaceum* [50]. The evaluation was repeated in at least three independent experiments and the results were averaged.

#### 2.5.6. Interaction with sortase A

The activity of sortase A (SrtA) was evaluated by using the SensoLyte^®^ 520 sortase A activity Assay Fluorimetric Kit (AnaSpec Inc., Fremont, CA, USA) in accordance with the manufacturer’s instructions. Compounds were dissolved in DMSO to get concentration 200 µg/mL. StrA was exposed to studied GTs and the results were collected after 10–70 min of ongoing incubation. The inhibitory activities of tested compounds against SrtA were determined as continuous recording of fluorescence intensity relative to the negative control (Tecan Infinite 200 Pro). The known SrtA inhibitor, 4-(hydroxymercuri)- benzoic acid (IC_50_ = 50.6 nM), was used as a positive control.

### 2.6. Statistical Analysis 

The experimental data were expressed as the mean ± standard deviation (±SD) of three independent experiments. The significance of differences between the means was evaluated by the OriginPro 8 software or Student’s *t*-test. Differences with *p* values < 0.05 were considered to be statistically significant.

## 3. Results and Discussion

### 3.1. Chemistry

The presence of multiple galloyl units in the GT molecules make them powerful antioxidants as well as effective antimicrobial agents. The strong contribution of the galloyl groups to these properties has been demonstrated several times, but it has also been observed that the carbohydrate moiety also plays an important role as well [21,51]. Carbohydrates are characterized by structural diversity and a multiplicity of nearly equivalent hydroxyl groups. As the 2-*C*-(hydroxymethyl)-branched-chain aldoses have several different hydroxyl groups, it was important to choose a suitable approach for the synthesis of their galloyl-esters. The synthetic method for the functionalization of branched aldoses is based on the modifications of primary hydroxyls, which tend to react faster, when doing this it is also necessary to protect secondary hydroxyls in order to carry out selective transformation of these polyfunctional molecules. For that reason, 2,3-*O*-isopropylidene derivatives of the 2-*C*-(hydroxymethyl)-branched aldoses were chosen as suitable compounds. This leaves the 2-*C*-hydroxymethyl branch free for galloylation, while keeping aldose in its furanose form.

**Scheme 1:**
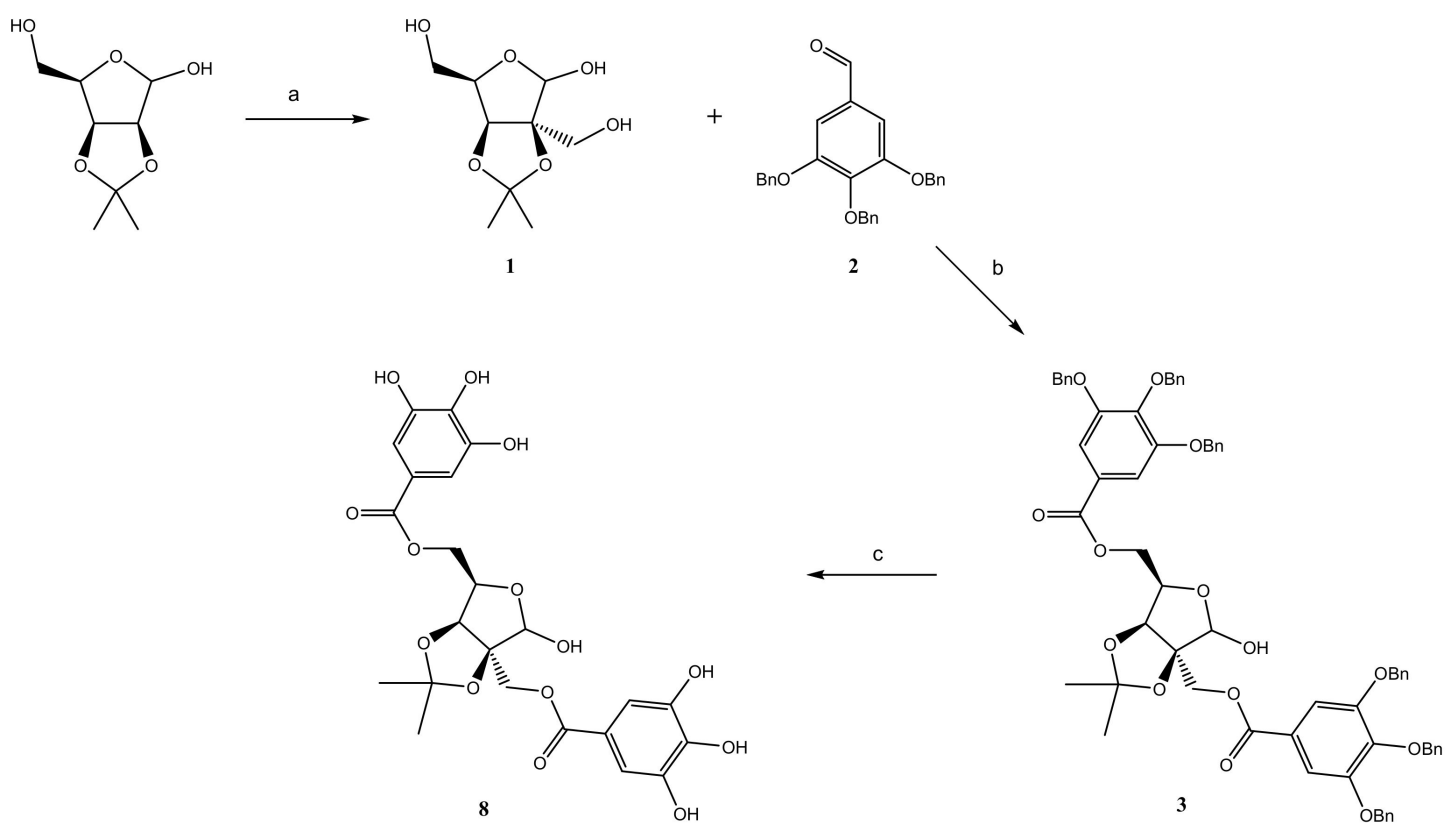
Synthesis of 2′,5-di-*O*-galloyl-2-*C*-(hydroxymethyl)-2,3-*O*-isopropylidene-d-lyxofuranose (**8**). *Reagents and conditions*: (**a**) 37% aq. CHO, K_2_CO_3_, methanol, argon atmosphere, reflux, 40 h (**b**) DCC, DMAP in DCM, reflux, 10 h (**c**) 10% Pd/C in methanol, H_2_ atmosphere, r.t., 16 h.

During the synthesis, all phenolic hydroxyl groups of GA were protected by benzylation to avoid intra- and inter-molecular stacking interactions between galloyl units. Moreover, the benzyl group is most frequently used for such purposes because it allows for quantitative removal by hydrogenation and a simple workup procedure. The *O*-isopropylidene derivatives, having distinct reactive hydroxyl groups, were used to introduce galloyl groups to the target sites of the sugar molecule. Hence, the appropriately protected 2-*C*-(hydroxymethyl)-branched aldose was esterified with **2** in the presence of DMAP as a catalyst and DCC as a coupling reagent, in order to obtain 2,3,4-tri-*O*-benzyl-galloylated 2-*C*-(hydroxymethyl)-branched aldoses **3**–**6** and the 2,3,4-tri-*O*-benzyl-galloyl derivative of d-fructose (**7**) in very good yields (69–84%). Subsequent debenzylation of compounds **3**–**7** led to the expected galloylated branched-chain aldoses **8**–**11** and galloylated d-fructose (**12**) in excellent yields (89–95%). A representative procedure for the synthesis of the 2′,5-di-*O*-galloyl-2-*C*-(hydroxymethyl)- 2,3-*O*-isopropylidene-d-lyxofuranose (**8**, G_2_Lyx) is depicted in Scheme 1. The structures of the new derivatives were determined on the basis of NMR spectroscopy and other analytical methods.

### 3.2. Antioxidant Activity

**Figure 1:**
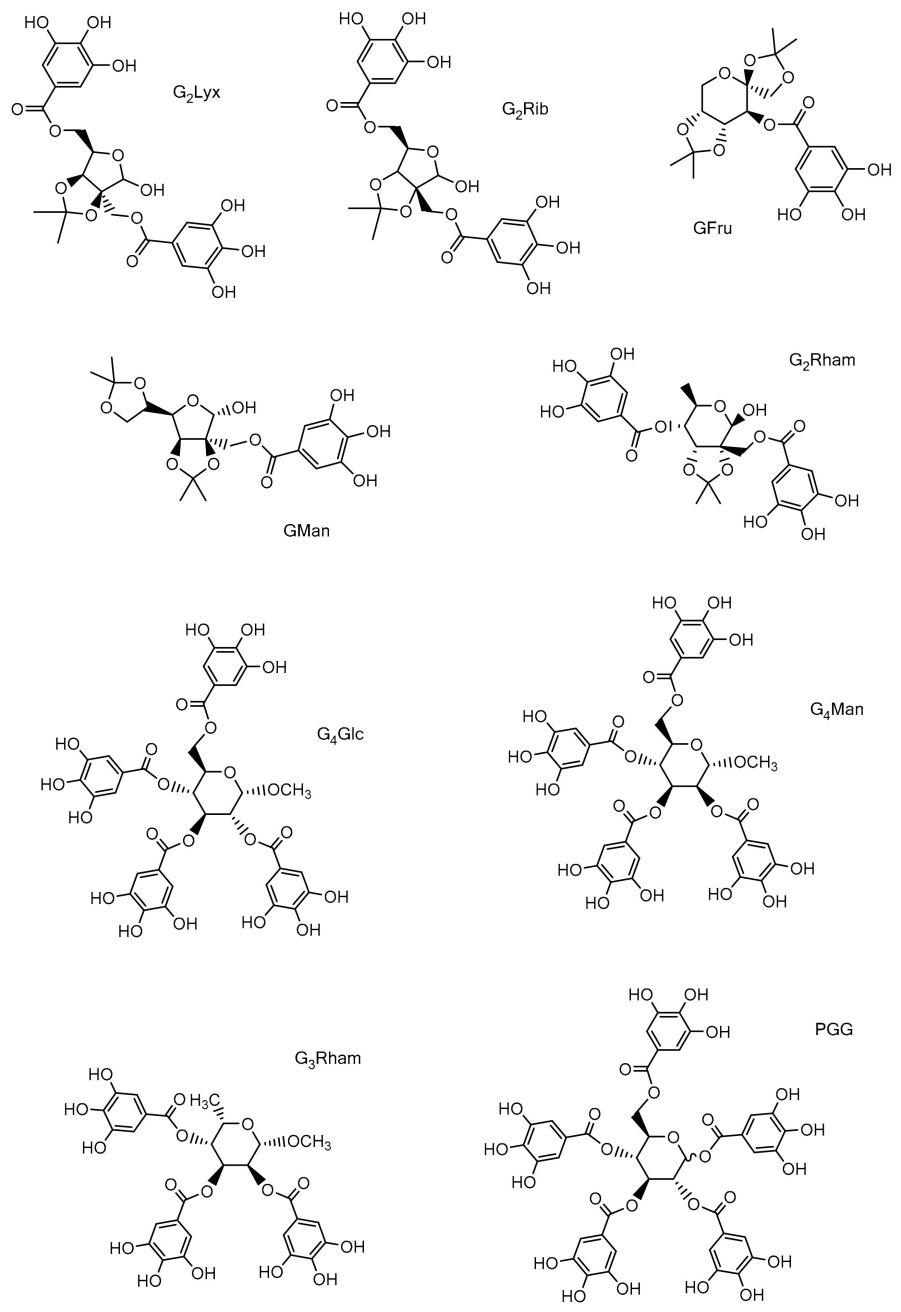
Studied compounds: 2′,5-di-*O*-galloyl-2-*C*-(hydroxymethyl)-2,3-*O*-isopropylidene- d-lyxose (G_2_Lyx); 2′,5-di-*O*-galloyl-2-*C*-(hydroxymethyl)-2,3-*O*-isopropylidene-d-ribose (G_2_Rib); 2′,4-di-*O*-galloyl-2-*C*-(hydroxymethyl)-2,3-*O*-isopropylidene-l-rhamnose (G_2_Rham); 2′-*O*-galloyl- 2-*C*-(hydroxymethyl)-2,3:5,6-di-*O*-isopropylidene-d-mannose (GMan); 3-*O*-galloyl- 1,2:4,5-di-*O*-isopropylidene-d-fructose (GFru); methyl 2,3,4,6-tetra-*O*-galloyl-α-d-glucoside (G_4_Glc); methyl 2,3,4,6-tetra-*O*-galloyl-α-d-mannoside (G_4_Man); methyl 2,3,4-tri-*O*-galloyl- α-l-rhamnoside (G_3_Rham); 1,2,3,4,6-penta-*O*-galloyl-d-glucose (PGG).

The antioxidant activity of compounds is related to their redox properties, which allow them to scavenge free radicals by acting as hydrogen donors or reducing agents. Antioxidant compounds usually contain aromatic rings that enable them to donate protons to free radicals formed during oxidation. A series of structurally different GTs (Figure 1), were screened for their free-radical-scavenging effect and reducing ability using the DPPH and FRAP assays. It is known that GA is a strong antioxidant due to the presence of three hydroxyl groups on the aromatic ring [52]. Thus, the high antioxidant efficiency of these compounds could be attributed to the presence of multiple galloyl groups in their structures. The experimental results indicated that the studied GTs exhibited notably different and concentration-dependent DPPH radical-scavenging effects (Table 1). Compounds PGG, G_4_Man, G_3_Rham, and G_4_Glc exhibited the highest radical-scavenging activity (94–98%), whereas the di-galloylated 2-*C*-(hydroxymethyl)- branched aldoses (G_2_Rib, G_2_Lyx, and G_2_Rham) manifested a slightly lower effect (85–88%). Moreover, as expected, mono-galloylated derivatives (GMan and GFru) displayed only moderate DPPH radical-scavenging activity (71–73%). Almost equal antioxidant activity was observed for PGG (98%) and G_4_Glc (96%), where the latter differs from the former by one less galloyl group. Moreover, comparable radical-scavenging activity was also observed for the compounds G_4_Man (95%) and G_4_Glc (96%), which have very similar molecular structures. However, in the case of G_3_Rham (94%) and the derivative G_2_Rham (85%), a noticeable decrease of antioxidant ability was observed. These compounds differ only by one galloyl group, but the lower antioxidant effect of G_2_Rham is probably influenced by the presence of isopropylidene groups in the molecule.

The variation in the radical-scavenging effect amongst the tested GTs was probably due to the different stability of the resulting oxygen-centred radical formed in these compounds. It was observed that the presence of isopropylidene groups in galloylated branched-chain aldoses (G_2_Rib, G_2_Lyx, and G_2_Rham) resulted in a decrease of antioxidant activity. The ability of studied GTs to quench DPPH radicals increased in the following order: GMan < GFru < G_2_Rham < G_2_Lyx ~ G_2_Rib ~ GA < G_3_Rham ~ G_4_Man ~ G_4_Glc ~ PGG.

The reduction of metal ions is used as an indicator of the electron-donating activity of compounds. The studied GTs exhibited varying ability to reduce Fe^3+^/ferricyanide complex to Fe^2+^/ferrocyanide, as seen in Table 1. Increases of the reducing power were correlated with the concentration of the tested compound, as well as with the number and substitution pattern of the galloyl groups attached to the carbohydrate core. The compounds PGG and unnatural GTs (G_4_Glc, G_4_Man, and G_3_Rham) exhibited remarkable potency for donating electrons to reactive free radicals, transforming them into more stable species. However, the presence of isopropylidene groups in galloylated 2-*C*-(hydroxymethyl)-branched aldoses (G_2_Rib, G_2_Lyx, and G_2_Rham) resulted in a noticeable decrease of their reducing power. The weakest reducing power (even lower than GA) was observed in the case of the derivatives GMan and GFru, each bearing only one galloyl moiety and two isopropylidene groups in their structure. The reducing power of the studied GTs increased in the following order: GFru < GMan < G_2_Lyx ~ G_2_Rham ~ GA ~ G_2_Rib < G_3_Rham ~ G_4_Man ~ G_4_Glc ~ PGG.

The experimental data thus revealed that the majority of the studied compounds exhibit an excellent radical-scavenging and reducing activity. The results (Table 1) demonstrated that outcomes from the FRAP assay are in good agreement with those of the DPPH assay, and that the overall antioxidant activity of each tested GT can be regarded as the sum of contributions of all the structural features in the molecule, depending on the number of galloyl groups and type of carbohydrate core, and the individual galloyl groups’ contributions varying with their position and spatial arrangement. Our findings are consistent with the previous reports on antioxidant potential of phenolic acids published by other authors [52,53].

### 3.3. Antibacterial and Antimycobacterial Activity

The antibacterial activity of the unnatural GTs was tested against *S. aureus* ATCC 29213, three MRSA isolates, *E. faecalis* ATCC 29212, and three VRE isolates. For screening of antimycobacterial effects a virulent isolate of *M. tuberculosis* and two non-tuberculous mycobacteria were used. The summary of results from experiments is presented in Table 2.

It can be concluded that most of the tested compounds were ineffective against both staphylococci and enterococci (> 128 µg/mL). PGG and unnatural epimeric GTs (G_4_Man and G_4_Glc) showed the highest potency (in the range of 16–64 µg/mL) within the tested series of compounds; however, it is still a moderate effect. In addition, the activity of these derivatives against enterococci is insignificant. Thus, the activity was not significantly affected by the presence of the mecA gene (in MRSA clinical isolates) or vanA (VRE clinical isolates). It can be summarized that unnatural G_3_Rham, galloylated 2-*C*-(hydroxymethyl)-branched aldoses (G_2_Rib, G_2_Lyx, G_2_Rham, and GMan), and mono-galloyl fructose (GFru) with isopropylidene groups in their structures were less active than PGG, G_4_Glc, and G_4_Man (Table 2). The antimycobacterial activity of the tested GTs was also insignificant. The compounds PGG, G_4_Glc, and G_4_Man showed only moderate antimycobacterial effect against *M. tuberculosis* (MIC ≥ 128 µg/mL). The galloylated 2-*C*-(hydroxymethyl)-branched aldoses (G_2_Rib, G_2_Lyx, G_2_Rham, and GMan), bearing isopropylidene groups did not show any antimycobacterial activity.

Several studies with similar natural compounds can be found in the literature. The antistaphylococcal activity of Thai mango (*M. indica)* kernel extract, containing more than 60% PGG and less than 1% gallic acid and methyl gallate, was studied by Jiamboonsri et al. The antibacterial effect was caused by PGG (MIC = 160 µg/mL) and was comparable with our results [22]. The group of Chan et al. investigated the antistaphylococcal activity of galloyl-substituted flavonol-rhamnosides extracted from *Calliandra tergemina* leaves. Three compounds, kaempferol-3-*O*-(2,3,4-tri-*O*-galloyl)-α-l-rhamnopyranoside, quercetin-3-*O*-(3,4-di-*O*-galloyl)-α-l-rhamnopyranoside and quercetin-3-*O*-(2,3,4-tri-*O*-galloyl)- α-l-rhamnopyranoside, showed only insignificant activity against three MRSA isolates (MIC 256 µg/mL). Compounds bearing only one galloyl group showed no anti-MRSA activity. However, multiple esterification of l-rhamnose molecule increased the antibacterial effect. Scanning electron microscopy (SEM) studies revealed that these compounds interact with the bacterial membrane [54].

Derivatives of gallic acid are able to interact with different bacterial cell structures. As mentioned above, one of the mechanisms of action is the interaction with the plasma membrane [54]. This interaction leads to leakage of cell proteins, nucleic acids, or inorganic compounds. Disruption of the cell membrane subsequently causes cell shrinkage and cell lysis, resulting in cell death [54]. In addition, GTs are also able to chelate metal ions, such as copper and iron, and make them inaccessible to microorganisms. For example, *Terminalia chebula* extract, containing significant amount of 1,2,6-tri-*O*-galloyl-d-glucose, demonstrated antibacterial activity against multidrug-resistant uropathogens, which might be due to iron-complexing properties [55,56]. PGG itself is able to interact directly with the bacterial cell wall. Zhao et al. studied PGG activity against *Candida glabrata*. The compounds were 10 times more active than fluconazole. The antifungal effect was due to the direct interaction of PGG with the fungal cell wall, without any interaction with glucan synthase [24].

The studied PGG analogues and isopropylidene-substituted GTs are new compounds whose antibacterial activity has not yet been described in the literature. However, the presented data demonstrate that the multifaceted GT-induced effects strongly depend on the investigated bacterial strain as well as on the structure of the GTs.

### 3.4. Antibiofilm Activity

**Figure 2:**
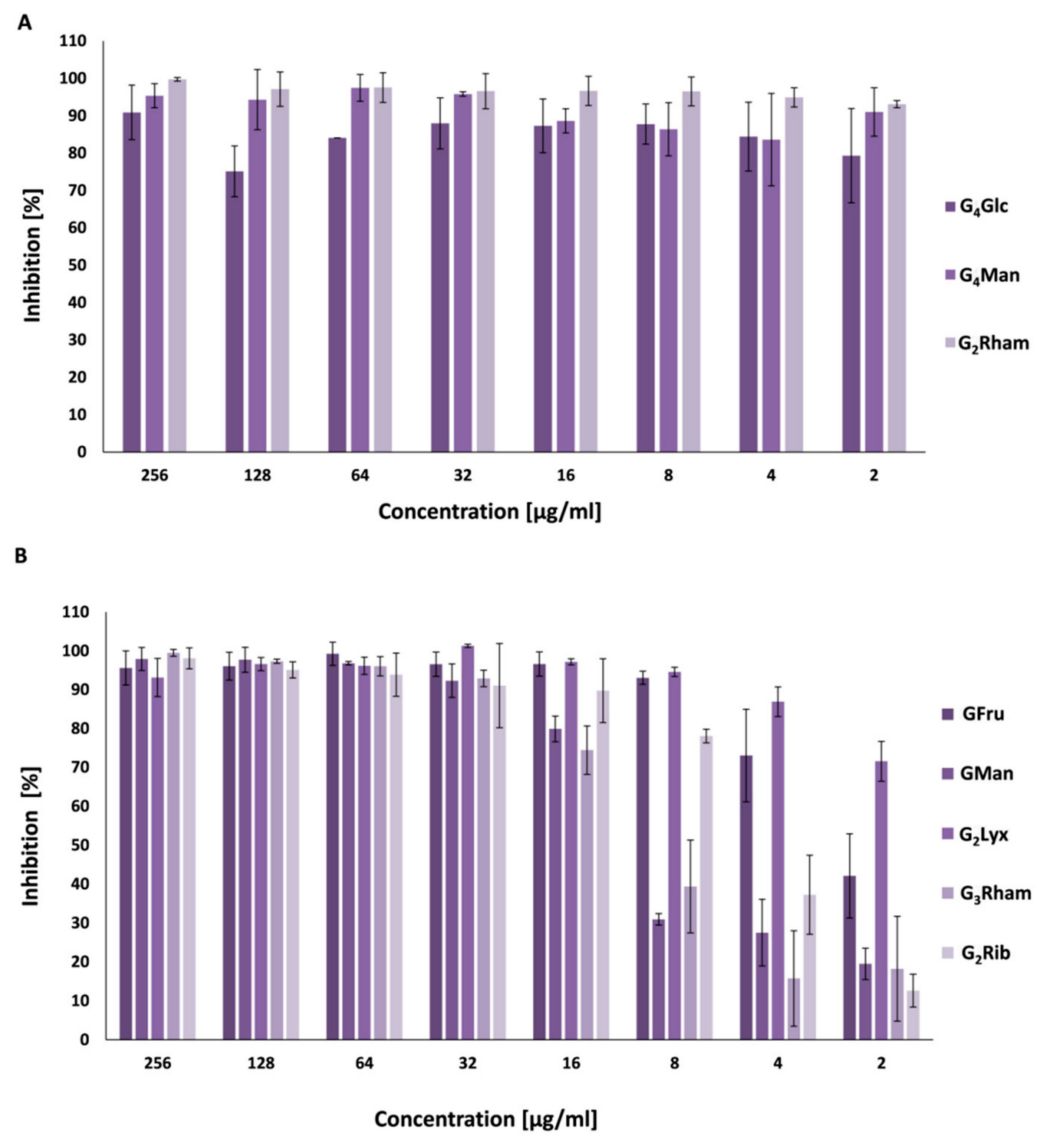
Inhibitory activities of compounds against *S. aureus* ATCC 29213 biofilm formation. *Compounds*: (**A**) methyl 2,3,4,6-tetra-*O*-galloyl-α-d-glucoside (G_4_Glc); methyl 2,3,4,6-tetra-*O*-galloyl- α-d-mannoside (G_4_Man); methyl 2,3,4-tri-*O*-galloyl-α-l-rhamnoside (G_3_Rham). (**B**) 3-*O*-galloyl- 1,2:4,5-di-*O*-isopropylidene-d-fructose (GFru); 2′-*O*-galloyl-2-*C*-(hydroxymethyl)-2,3:5,6-di-*O*-isopro- pylidene-d-mannose (GMan); 2′,5-di-*O*-galloyl-2-*C*-(hydroxymethyl)- 2,3-*O*-isopropylidene-d-lyxose (G_2_Lyx); 2′,4-di-*O*-galloyl-2-*C*-(hydroxymethyl)-2,3-*O*-isopropylidene- l-rhamnose (G_2_Rham); 2′,5-di-*O*-galloyl-2-*C*-(hydroxymethyl)-2,3-*O*-isopropylidene-d-ribose (G_2_Rib).

Bacterial biofilms protect the microbial community from external damage and increase the persistence of chronic infections. Selected compounds were examined as inhibitors and disruptors of *S. aureus* ATCC 29213 biofilms. The results indicate that both groups, PGG analogues (Figure 2A) and galloylated 2-*C*-(hydroxymethyl)-branched aldoses (Figure 2B), efficiently inhibited *S. aureus* biofilm formation at concentrations lower than MIC values. For example, the minimum biofilm inhibition concentration (MBIC) of compound G_4_Glc was 64 times lower than its MIC value, MBIC for G_4_Man was 32 times lower, and MBIC for G_3_Rham was 256 times lower than its MIC value. These results identify G_3_Rham as the most potent inhibitory compound from this series.

**Figure 3:**
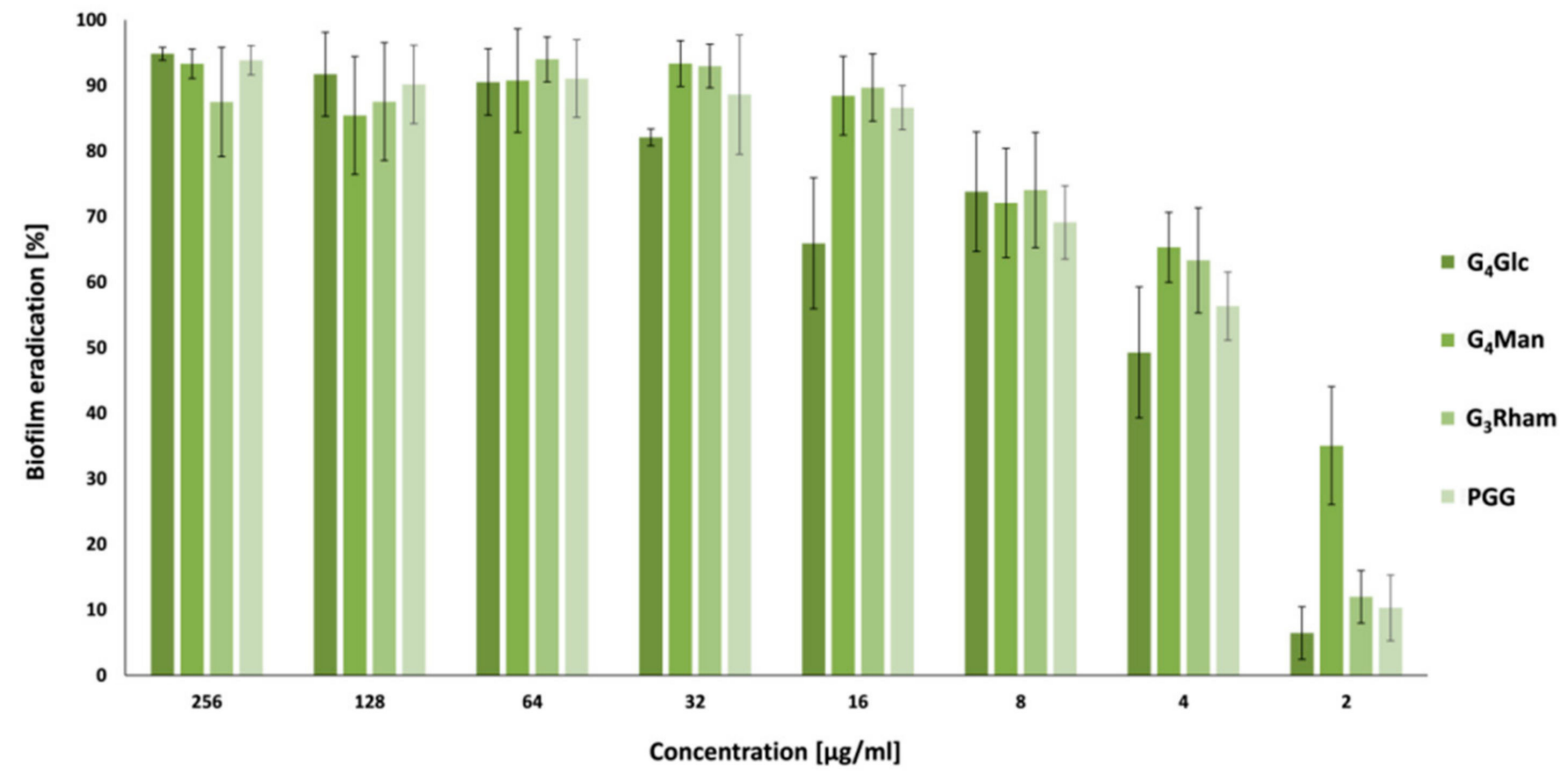
Eradication activities of compounds against *S. aureus* ATCC 29213 biofilm formation. *Compounds*: methyl 2,3,4,6-tetra-*O*-galloyl-α-d-glucoside (G_4_Glc); methyl 2,3,4,6-tetra-*O*-galloyl- α-d-mannoside (G_4_Man); methyl 2,3,4-tri-*O*-galloyl-α-l-rhamnoside (G_3_Rham) and 1,2,3,4,6-penta- *O*-galloyl-d-glucose (PGG).

New anti-biofilm compounds that can effectively eradicate bacterial biofilms are desirable to prevent various infections. Eradication activity was tested for PGG and its analogues G_4_Glc, G_4_Man and G_3_Rham. All tested compounds eliminated 80% of the matured biofilm of *S. aureus* at concentrations lower than the MIC value against planktonic cells. In this case, as well, G_3_Rham was the most active compound. G_3_Rham eradicated biofilms of *S. aureus* at a concentration 16 times lower than its MIC value (Figure 3). The interesting biofilm inhibitory and eradication activity against *S. aureus* make unnatural GTs excellent biofilm-controlling agents for healthcare and food processing facilities.

A number of structurally similar natural GTs with remarkable antimicrobial and antibiofilm properties have been studied. It was reported that extracts of *Cytinus* possess interesting antimicrobial activity against Gram-positive bacteria (*S. aureus*, *Staphylococcus epidermidis* and *Enterococcus faecium*). Characterization of the tannin profile of *Cytinus hypocistis* and *Cytinus ruber* revealed a significant number of GTs, in particular 1-*O*-galloyl-β-d-glucose and PGG. Three pathogenic species were sensitive to *Cytinus* extracts, and a significant inhibition of *S. epidermidis* biofilm formation was also observed [57]. Recently, Bag et al. published two studies investigating the antimicrobial activity of GT, 1,2,6-tri-*O*-galloyl-β-d-glucopyranose, isolated from *T. chebula,* against multidrug-resistant *E. coli*. This tri-*O*-galloyl derivative of d-glucose, was effective against multidrug-resistant uropathogens, and acted synergistically in combination with gentamicin and trimethoprim and additively in combination with amoxicillin, ciprofloxacin and ceftazidine. This substance was able to eradicate the preformed *E. coli* biofilms and at the same time act with synergistic antibiofilm activity in combination with gentamicin and trimethoprim [55,58]. This compound differs from the test compound G_4_Glc only by the number and position of the gallic acid residues in the molecule. Brackman et al. have shown that natural hamamelitannin (which is structurally very similar with test compound G_2_Rib) interfaces with QS in *S. aureus*, and thereby increases the susceptibility of *S. aureus* biofilms to various antibiotics [34].

Overall, our results demonstrate that GTs have sufficient inhibitory effect against *S. aureus* ATCC 29213 biofilm formation. This preliminary study suggests that the antibiofilm activity of studied compounds did not increase with an increasing number of galloyl units. Furthermore, the activity is affected by the sugar moiety. The most active antibiofilm compound, G_3_Rham, had one of the lowest activities against the planktonic cells of *S. aureus*. These experiments showed that the main mechanism of action against biofilm formation is not related to antibacterial activity against planktonic cells. However, as the number of the test compounds was limited, the structure-activity relationship cannot be discussed in more depth.

### 3.5. Quorum Sensing Inhibition

QS is a communication mechanism that regulates bacterial virulence in pathogens. A number of plant phenolics have been identified as important microbial growth and QS inhibitors [14,59]. It was expected that the compounds could work as inhibitors of the QS system, as they interacted with *S. aureus* biofilms at concentrations lower than MIC values. This activity was tested on the opportunistic bacterium *C. violaceum*. One particular characteristic of this genus is the synthesis of the purple pigment, violacein, which is regulated by QS. Inhibition of this system blocks violacein production and leads to the growth of white or colourless colonies of *C. violaceum.* Interestingly, all tested compounds, except for G_2_Rham, inhibited violacein production (Table 3). However, it was not possible to determine whether the observed effect was related to the sugar moiety or to the number of galloyl groups due to the limited number of the investigated compounds and their slightly different activities. Similarly, it is not possible to explain the complete inactivity of G_2_Rham. Nevertheless, it is important to be aware of the fact that QS active compounds have non-linear effects [60].

### 3.6. Inhibition of Sortase A

**Figure 4:**
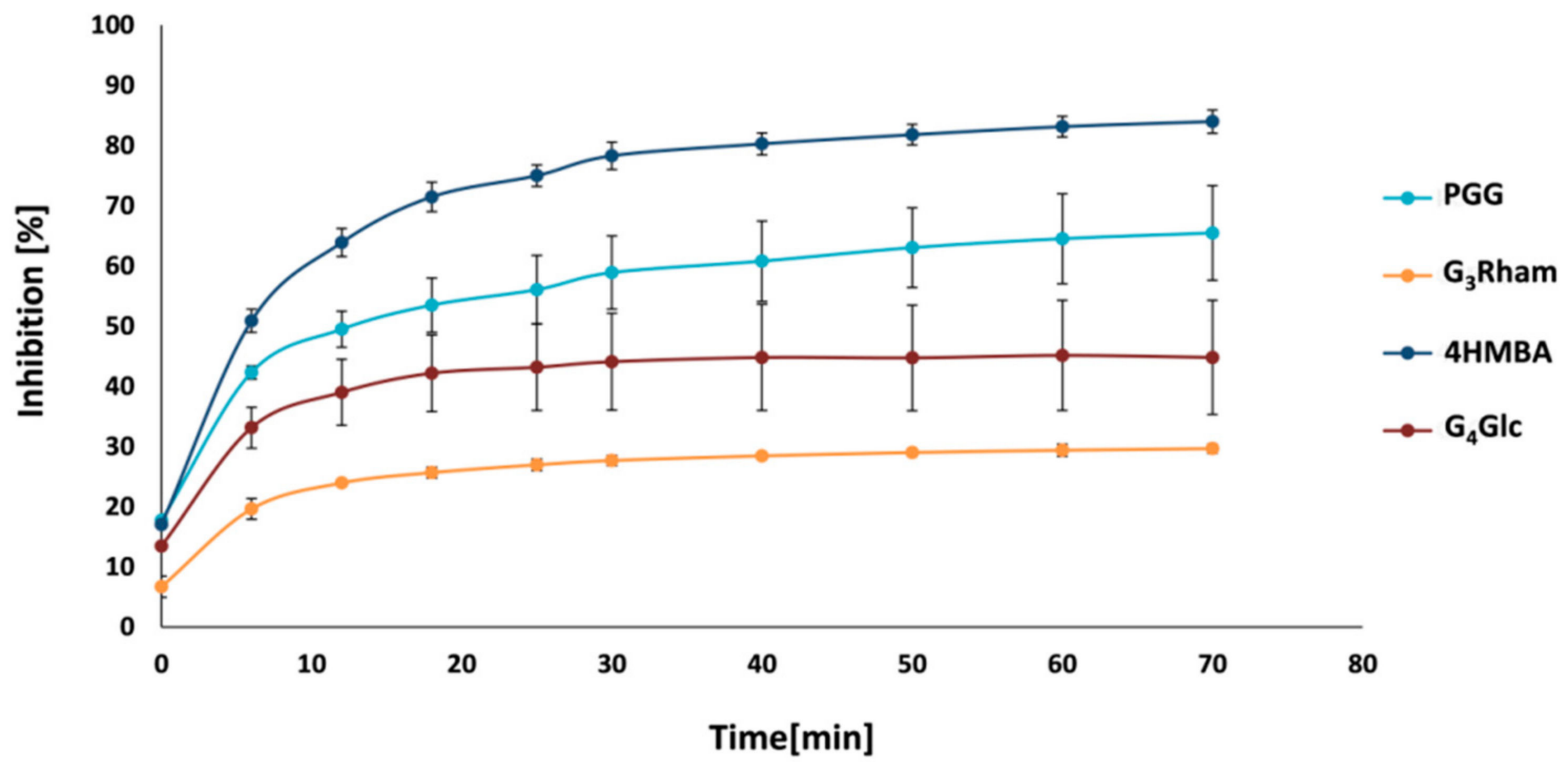
Inhibitory activity of compounds against SrtA. *Compounds*: 1,2,3,4,6-penta-*O*-galloyl- d-glucose (PGG); methyl 2,3,4-tri-*O*-galloyl-α-l-rhamnoside (G_3_Rham) and methyl 2,3,4,6-tetra-*O*-galloyl-α-d-glucoside (G_4_Glc); 4-(hydroxymercuri)benzoic acid (4HMBA).

Since SrtA, which anchors surface proteins to the cell wall, plays a critical role in Gram-positive bacterial pathogenesis, it should be possible to target bacterial virulence and treat infections by inhibiting the activity of SrtA. The structurally different compounds PGG, G_4_Glc and G_3_Rham were investigated as potential inhibitors of SrtA. The experiments indicated that these compounds are able to block the pathogenic action of SrtA, and the degree of enzyme inhibition is plotted against time in Figure 4. PGG (bearing five galloyl units in its structure) exhibited the most potent inhibitory effect against SrtA among all tested compounds. It inhibited SrtA by 65.5 ± 7.9% after 70 min. The tetra- and tri-*O*-galloylated compounds G_4_Glc and G_3_Rham had approximately half (44.8 ± 9.5%) and quarter inhibitory activity (29.6 ± 0.8%), respectively. These findings indicate that the ability to inhibit SrtA increases with the degree of galloylation. Thus, the number of galloyl groups attached to the carbohydrate core seems to be a critical factor.

The ability of PGG derivatives to inhibit SrtA has not been described in the literature yet. However, there are many studies examining the effect of other polyphenolic compounds, mainly flavonoids, on bacterial sortases. Wang et al. showed that the flavonoid baicalin binds to sortase B in *S. aureus* and reduces its virulence [61]. Other flavonoids, especially kurarinol, isolated from the root of *Sophora flavescens*, acted as inhibitors of SrtA as well. It was observed that the presence of prenyl groups in kurarinol was essential for its strong inhibitory potency [62]. Other sortase inhibitors, which are potentially useful in the treatment of bacterial infections, include isovitexin [63], astilbin [64], and rutin [65].

The findings described above suggest that PGG and its structural analogues G_4_Glc and G_3_Rham are able to inhibit the catalytic activity of SrtA in vitro, but have little effect on bacterial growth. The compounds interfered with the adhesion of *S. aureus* biofilm, and thus hold promise for the development of anti-virulence agents against medical device infections caused by *S. aureus*. It seems that unnatural GTs may be an important new direction in the field of research into SrtA inhibitors.

## 4. Conclusions

In summary, new GTs derived from 2-*C*-(hydroxymethyl)-branched aldoses were synthesized and characterized on the basis of their physical and spectral data. Evaluation of antioxidant, antimicrobial, and antibiofilm activity showed differences among the newly prepared derivatives and three synthetic analogues of PGG and commercial antioxidant gallic acid. Results from the present study indicate that unnatural GTs are promising antioxidants and radical-scavengers. The compounds PGG, G_4_Glc, and G_3_Rham inhibited SrtA, and it can be stated that the inhibitory effect increased with the increasing number of galloyl groups. On the other hand, the inhibitory effect was also dependent on the type of sugar. It can be hypothesized that the observed antibacterial activity is related to the inhibition of SrtA, although, there are other mechanisms which are also likely to be involved. All of the investigated GTs proved to be potent inhibitors and disruptors of *S. aureus* biofilms at concentrations much lower than the MIC values. The promising activity at inhibiting the growth of *S. aureus* biofilms makes GTs valuable alternatives to synthetic antioxidants and currently used antibiofilm agents suitable for application in the biomedical, pharmaceutical, cosmetics, consumer products, and food industries.

## Figures and Tables

**Table 1:** Antioxidant activity. DPPH radical-scavenging assay and reducing power of 2′,5-di-*O*-galloyl-2-*C*-(hydroxymethyl)-2,3-*O*-isopropylidene-d-lyxose (G_2_Lyx); 2′,5-di-*O*-galloyl- 2-*C*-(hydroxymethyl)-2,3-*O*-isopropylidene-d-ribose (G_2_Rib); 2′,4-di-*O*-galloyl-2-*C*-(hydroxymethyl)- 2,3-*O*-isopropylidene-l-rhamnose (G_2_Rham); 2′-*O*-galloyl-2-*C*-(hydroxymethyl)-2,3:5,6-di-*O*-isopro- pylidene-d-mannose (GMan); 3-*O*-galloyl-1,2:4,5-di-*O*-isopropylidene-d-fructose (GFru); methyl 2,3,4,6-tetra-*O*-galloyl-α-d-glucoside (G_4_Glc); methyl 2,3,4,6-tetra-*O*-galloyl-α-d-mannoside (G_4_Man); methyl 2,3,4-tri-*O*-galloyl-α-l-rhamnoside (G_3_Rham); 1,2,3,4,6-penta-*O*-galloyl-d-glucose (PGG).

Comp.	Concentration (mM)	DPPH Scavenging Activity (%)	Reducing Power (Absorbance)
**G_2_Lyx**	0.1	49.97 ± 1.14	0.675 ± 0.041
	0.25	63.98 ± 1.05	0.886 ± 0.015
	0.5	75.35 ± 1.61	1.263 ± 0.053
	1	87.36 ± 1.35	1.699 ± 0.082
**G_2_Rib**	0.1	52.67 ± 1.27 *	0.698 ± 0.028
	0.25	67.55 ± 1.08 *	0.979 ± 0.015
	0.5	78.39 ± 1.11	1.185 ± 0.031
	1	87.95 ± 1.39	1.761 ± 0.102
**G_2_Rham**	0.1	49.68 ± 2.13	0.622 ± 0.036
	0.25	67.04 ± 1.72	0.904 ± 0.045
	0.5	79.63 ± 1.21	1.325 ± 0.018
	1	85.18 ± 1.03	1.711 ± 0.072
**GMan**	0.1	33.57 ± 1.66 ***	0.534 ± 0.081 *
	0.25	48.19 ± 2.09 **	0.871 ± 0.016 *
	0.5	68.42 ± 1.74 *	1.054 ± 0.037 *
	1	71.28 ± 1.16 **	1.128 ± 0.057 **
**GFru**	0.1	27.67± 1.25 ***	0.485 ± 0.044 *
	0.25	39.29 ± 1.08 ***	0.621 ± 0.073 **
	0.5	62.18 ± 1.13 ***	0.823 ± 0.051 **
	1	73.36± 2.19 ***	1.105 ± 0.069 **
**G_4_Glc**	0.1	69.72 ± 1.59 ***	0.826 ± 0.017 *
	0.25	75.35 ± 1.98 **	1.297 ± 0.044 **
	0.5	84.49 ± 2.27 *	1.999 ± 0.081 **
	1	95.96 ± 1.77 **	2.267 ± 0.086 **
**G_4_Man**	0.1	68.19 ± 1.54 ***	0.779 ± 0.051
	0.25	77.02 ± 2.11 **	1.307 ± 0.079 ***
	0.5	83.76 ± 1.99 *	1.878 ± 0.068 **
	1	95.01 ± 1.63 **	2.238 ± 0.039 **
**G_3_Rham**	0.1	71.38 ± 1.74 ***	0.801 ± 0.073 *
	0.25	78.14 ± 1.49 **	1.464 ± 0.057 **
	0.5	86.63 ± 2.08 *	1.935 ± 0.108 ***
	1	94.13 ± 1.27 **	2.197 ± 0.096 **
**PGG**	0.1	75.32 ± 2.01 ***	0.918 ± 0.025 *
	0.25	80.27 ± 1.14 ***	1.506 ± 0.037 **
	0.5	92.35 ± 1.58 **	2.104 ± 0.093 ***
	1	97.88 ± 1.22 **	2.307 ± 0.034 **
**GA**	0.1	47.56 ± 1.45	0.688 ± 0.015
	0.25	63.03 ± 2.06	1.002 ± 0.071
	0.5	76.89 ± 1.73	1.289 ± 0.059
	1	88.14 ± 1.16	1.736 ± 0.084

Gallic acid (GA) was used as positive control. Data represent the mean values ± SD of three independent experiments; * *p* < 0.05, ** *p* < 0.01, *** *p* < 0.001 indicate significant differences in comparison with positive control.

**Table 2:** Antimicrobial activities of investigated compounds against Gram-positive bacteria and mycobacteria expressed as a minimum inhibitory concentration (MIC [µg/mL]/[mM]) compared to gallic acid (GA), ampicillin (AMP) and isoniazid (INH).

Comp.	MIC ([µg/mL]/[mM])										
	SA	MRSA-1	MRSA-2	MRSA-3	EF	VRE-1	VRE-2	VRE-3	MT	MK	MS
**G_4_Glc**	256/ 318	256/ 318	**64/** **79.7**	**64/** **79.7**	**32/** **39.8**	**64/** **79.7**	256/ 318	256/ 318	>128/ >159	256 318	256/ 318
**G_4_Man**	64/ 79.7	256/ 318	**64/** **79.7**	**64/** **79.7**	**16/** **19.9**	**32/** **39.8**	256/ 318	256/ 318	>128/ >159	128/ 159	256/ 318
**G_3_Rham**	128/ 201	256/ 403	128/ 201	128/ 201	**32/** **50.4**	128/ 201	256/ 403	256/ 403	>128/ >201	128/ 201	256/ 403
**G_2_Lyx**	>256/ >504	>256/ >504	>256/ >504	>256/ >504	>256/ >504	>256/ >504	>256/ >504	>256/ >504	>128/ >252	>256/ 504	>256/ 504
**G_2_Rham**	256/ 456	256/ 456	256/ 456	256/ 456	>256/ >456	>256/ >456	>256/ >456	>256/ >456	>256/ >456	>256/ >456	>256/ >456
**G_2_Rib**	128/ 244	256/ 488.2	256/ 488	128/ 244	>256/ >488	>256/ >488	>256/ >488	>256/ >488	>128/ >244	>256/ >488	>256/ >488
**GMan**	128/ 289	256/ 578	256/ 578	128/ 289	>256/ >578	>256/ >578	>256/ >578	>256/ >578	>128/ >289	>256/ >578	>256/ >578
**GFru**	128/ 310	128/ 310	256/ 620	128/ 310	>256/ >620	>256/ >620	>256/ >620	>256/ >620	>128/ >310	>256/ >620	>256/ >620
**PGG**	**32/** **34.0**	128/ 136	**32/** **34.0**	**16/** **17.0**	**16/** **17.0**	**64/** **68.0**	128/ 136	256/ 272	>128/ >136	256/ 272	>256/ >272
**GA**	256/ 1487	64/ 371	64/ 371	32/ 185	>256/ >1487	>256/ >1487	>256/ >1487	>256/ >1487	>128/ >743	256/ 1487	>256/ >1487
**AMP**	2/ 5.72	16/ 45.8	>16/ >45.8	>16/ >45.8	1/ 2.86	4/ 11.5	4/ 11.5	4/ 11.5	–	–	–
**INH**	–	–	–	–	–	–	–	–	8 58.0	4 29.1	16 117

*Staphylococcusaureus* ATCC 29213 (SA); MRSA1–3 (clinical isolates of methicillin-resistant *S. aureus* 63718, SA 630, and SA 3202; National Institute of Public Health, Prague, Czech Republic); *Enterococcus faecalis* ATCC 29213 (EF), and vancomycin-resistant enterococci VRE 1–3 (VRE 342B, VRE 368, VRE 725B) [43]; *Mycobacterium tuberculosis* H_37_Ra/ATCC 25177 (MT); *Mycobacterium kansasii* DSM 44162 (MK); *Mycobacterium smegmatis* ATCC 700084 (MS). *Compounds*: methyl 2,3,4,6-tetra- *O*-galloyl-α-d-glucoside (G_4_Glc); methyl 2,3,4,6-tetra-*O*-galloyl-α-d-mannoside (G_4_Man); methyl 2,3,4-tri-*O*-galloyl- α-l-rhamnoside (G_3_Rham); 2′,5-di-*O*-galloyl-2-*C*-(hydroxymethyl)-2,3-*O*-isopropylidene-d-lyxose (G_2_Lyx); 2′,4-di-*O*-galloyl- 2-*C*-(hydroxymethyl)-2,3-*O*-isopropylidene-l-rhamnose (G_2_Rham); 2′,5-di-*O*-galloyl-2-*C*-(hydroxymethyl)- 2,3-*O*-isopropylidene-d-ribose (G_2_Rib); 2′-*O*-galloyl-2-*C*-(hydroxymethyl)-2,3:5,6-di-*O*-isopropylidene-d-mannose (GMan); 3-*O*-galloyl-1,2:4,5-di-*O*-isopropylidene-d-fructose (GFru); 1,2,3,4,6-penta-*O*-galloyl-d-glucose (PGG).

**Table 3:** Inhibitory activity of compounds to QS system of *C. violaceum*.

Comp. (conc. 10 mg/mL)	Diameter of Inhibition Zone on Agar [mm]
**G_4_Glc**	5.00 ± 0.01
**G_4_Man**	8.50 ± 2.90
**G_3_Rham**	7.00 ± 1.40
**G_2_Rham**	–
**G_2_Rib**	8.30 ± 0.62
**G_2_Lyx**	5.00 ± 1.01
**GMan**	3.00 ± 0.02
**GFru**	6.50 ± 1.31
**PGG**	6.00 ± 0.01

*Compounds*: methyl 2,3,4,6-tetra-*O*-galloyl-α-d-glucoside (G_4_Glc); methyl 2,3,4,6-tetra-*O*-galloyl- α-d-mannoside (G_4_Man); methyl 2,3,4-tri-*O*-galloyl-α-l-rhamnoside (G_3_Rham); 2′,4-di-*O*-galloyl- 2-*C*-(hydroxymethyl)-2,3-*O*-isopropylidene-l-rhamnose (G_2_Rham); 2′,5-di-*O*-galloyl-2-*C*-(hydroxy- methyl)-2,3-*O*-isopropylidene-d-ribose (G_2_Rib); 2′,5-di-*O*-galloyl-2-*C*-(hydroxymethyl)-2,3-*O*-iso- propylidene-d-lyxose (G_2_Lyx); 2′-*O*-galloyl-2-*C*-(hydroxymethyl)-2,3:5,6-di-*O*-isopropylidene- d-mannose (GMan); 3-*O*-galloyl-1,2:4,5-di-*O*-isopropylidene-d-fructose (GFru) and 1,2,3,4,6-penta-*O*-galloyl-d-glucose (PGG).

## Data Availability

Data is contained within the article.
